# Automated region of interest retrieval and classification using spectral analysis

**DOI:** 10.1186/1746-1596-3-S1-S17

**Published:** 2008-07-15

**Authors:** Myriam Oger, Philippe Belhomme, Jacques Klossa, Jean-Jacques Michels, Abderrahim Elmoataz

**Affiliations:** 1 TRIBVN, 39 rue Louveau, 92320 Châtillon, France; 2 EPF Ecole d'ingénieurs, 3 bis rue Lakanal, 92330 Sceaux, France; 3 Histo-imagerie quantitative, GRECAN (EA 1772, IFR 146 ICORE University of Caen Basse-Normandie), F. Baclesse Cancer Centre, Bd Général Harris, F 14076 Caen, France; 4 Service d'Anatomie pathologique, F. Baclesse Cancer Centre, Bd Général Harris, F 14076 Caen, France; 5 GREYC, UMR 6072, University of Caen Basse-Normandie, 6 Boulevard du Maréchal Juin, 14050 Caen Cedex, France

## Abstract

Efficient use of whole slide imaging in pathology needs automated region of interest (ROI) retrieval and classification, through the use of image analysis and data sorting tools. One possible method for data sorting uses Spectral Analysis for Dimensionality Reduction. We present some interesting results in the field of histopathology and cytohematology.

In histopathology, we developed a Computer-Aided Diagnosis system applied to low-resolution images representing the totality of histological breast tumour sections. The images can be digitized directly at low resolution or be obtained from sub-sampled high-resolution virtual slides. Spectral Analysis is used (1) for image segmentation (stroma, tumour epithelium), by determining a «distance» between all the images of the database, (2) for choosing representative images and characteristic patterns of each histological type in order to index them, and (3) for visualizing images or features similar to a sample provided by the pathologist.

In cytohematology, we studied a blood smear virtual slide acquired through high resolution oil scanning and Spectral Analysis is used to sort selected nucleated blood cell classes so that the pathologist may easily focus on specific classes whose morphology could then be studied more carefully or which can be analyzed through complementary instruments, like Multispectral Imaging or Raman MicroSpectroscopy.

## Introduction

Efficient use of whole slide imaging (WSI) in Pathology needs an automated region of interest (ROI), retrieval and classification; this can be achieved through the use of image segmentation and data sorting tools. The present paper aims at illustrating, through two examples, the power of spectral analysis, which can be used alone or in addition to image segmentation for data reduction, feature classification as well as image visualisation.

## Materials and methods

### Material

The first application concerns 73 WSI of HES stained histological sections of breast tumours recorded at a resolution of 6.3 *μ*m/pixel. The second one concerns a WSI of MG stained blood smear recorded at a resolution of 0.17 *μ*m/pixel using an Aperio slide scanner.

A minimal segmentation was performed to isolate breast tumour tissue or to eliminate erythrocytes from blood smear.

### Principle of spectral analysis

The main point of this technique is to introduce a useful metric on data set based on the connectivity of points within the graph of data, and also provide coordinates on the data set that reorganize the points according to this metric [1,2]. Let *X *= {*x*_*1*_,*x*_*2*_,...,*x*_*N*_} be *N *data points (images), each data *x*_*i*_ϵ*R*^*n*^ where *n *is the dimension of the space data (measures). The first step is to represent the dataset *X *= {*x*_*1*_,*x*_*2*_,...,*x*_*N*_} by a weighted symetric graph *G *= (*V*, *E*) where each data point *x*_*i *_corresponds to a node. Two nodes *x*_*i *_and *x*_*j *_are connected by an edge with weight w(x_i_,x_j_) = w(x_j_,x_i_), reflecting the degree of similarity (or affinity) between these two points. The weight *w*(.,.) describes the first-order interaction between the data points and its choice is application-driven. For instance, in applications where a distance *d*(.,.) already exists on the data, it is custom to weight the edge between *x*_*i *_and *x*_*j *_by:

*w*(*x*_*i*_, *x*_*j*_) = exp(-*d*(*x*_*i*_, *x*_*j*_)^2^/*ε*)

where *ε *> 0 is a scale parameter, while other weighting functions can be also used.

Following a classical construction in spectral graph theory and manifold learning, we now create a random walk on the data set *X *by forming the kernel:

$$p\left(x_{i},x_{j}\right)=\frac{w\left(x_{i},x_{j}\right)}{d\left(x_{i}\right)}$$

Where:

$$d(x_{i})=\sum_{x_{k}\in X}w(x_{i,}x_{k})$$

is the degree of node *x*_*i*_.

As we have that *p*(*x*_*i*_, *x*_*j*_) ≥ 0 and

$$\sum_{x_{j}\in X}p(x_{i,}x_{j})=1$$

the quantity *p*(*x*_*i*_, *x*_*j*_) ≥ 0 can be interpreted as the probability of random walker to jump from *x*_*i *_to *x*_*j *_in single time step.

From spectral theory and harmonic analysis we know that the eigenfunctions can be interpreted as a generalization of the Fourier harmonics on the manifold defined by the data points. In our problem, smaller eigenvalues correspond to higher frequency eigenfunctions, and larger eigenvalues correspond to lowers ones.

The eigenvalues and eigenvectors provide embedding coordinates for the set *X*. The data points can be mapped into Euclidean space via embedding:

Ψ_*t *_→ (*ψ*_1_(*x*), *ψ*_2_(*x*),..., *ψ*_*m*(*t*)_(*x*)).

The second eigenvector *ψ*_2 _is known as the Fiedler vector and can be used to order the underlying dataset *X *(segmentation and data reduction). When it is associated with the third eigenvector *ψ*_3_, it allows a visualization of the base.

## Results

### Breast cancer

**Figure 1:**
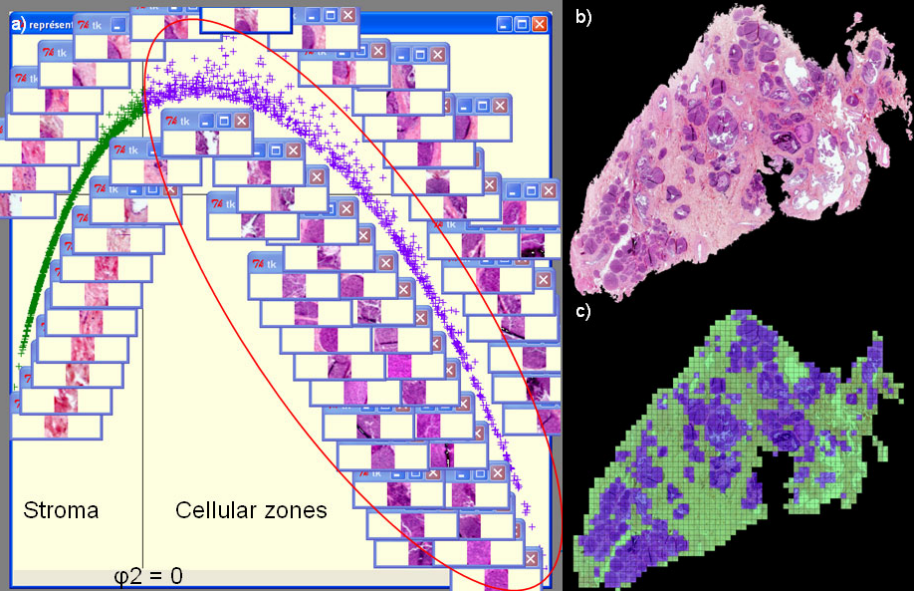
Result of breast VS segmentation by spectral analysis: (a) visualization of data sorting allowing segmentation of (b) the original image, (c) result of the segmentation.

**Figure 2:**
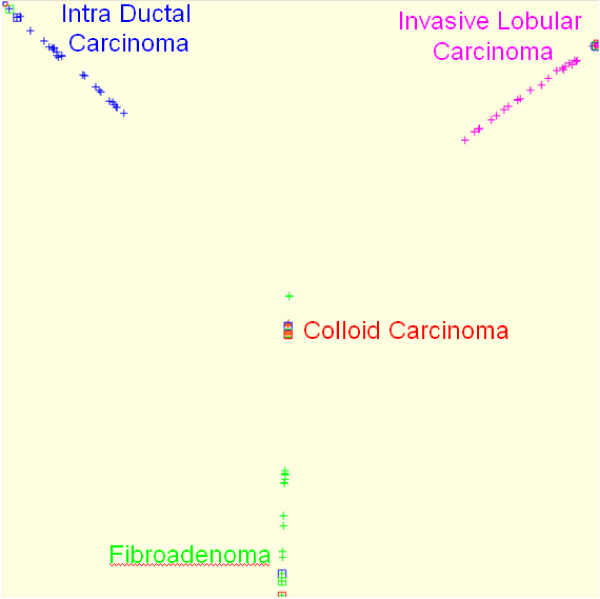
Selection of the most representative (□) epithelial zone patches of each histological type.

**Figure 3:**
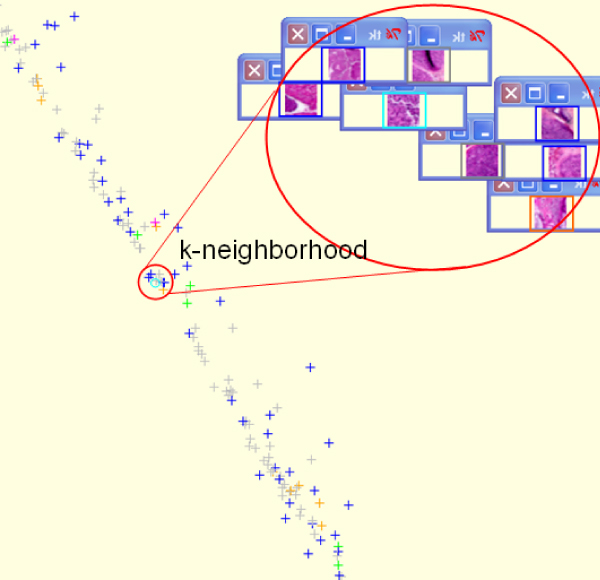
Visualization of each patch and its neighbourhood in order to exhibit the most similar patches.

In this case, the *ψ*_2 _eigenvector was used to segment tumour tissue into two classes: stroma and epithelial zones (Figure 1). The method has been applied to all the images of the database. Then spectral analysis was used to select the most representative epithelial zone patches of each histological type (Figure 2). Finally, *ψ*_2 _and *ψ*_3 _allow a data sorting and a visualization of each patch and its neighbourhood in order to present the most similar patches (Figure 3).

### Blood smears

**Figure 4:**
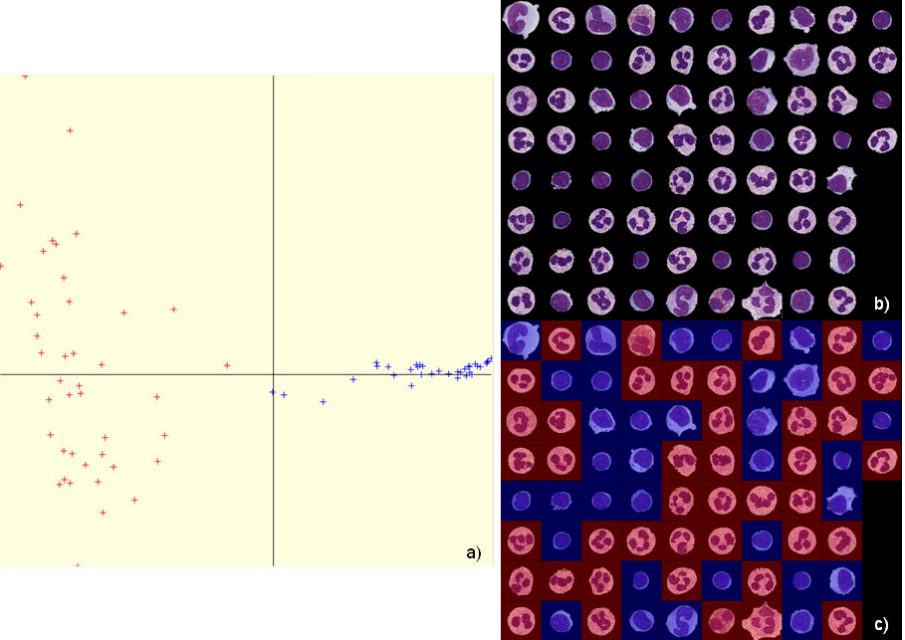
Result of isolated white blood cell base "segmentation" by spectral analysis: (a) visualization of the data sorting (lymphocytes are shown in blue) allowing the partition of (b) the base of isolated cells, (c) view of isolated cells sorted by spectral analysis.

For this application, spectral analysis was used to "segment", by data sorting, the image base of isolated blood cells into two classes: polymorphonuclear cells and lymphocytes (Figure 4).

## Conclusion

Spectral Analysis is a promising approach for computer aided diagnosis of cancers (automated global analysis of histological tumour sections) as well as for automated sorting of isolated cells. The resulting concentration of objects of interest allows the pathologist to focus on specific regions whose morphology can be further studied more carefully or analyzed through complementary instruments, like Multispectral Imaging or Raman spectroscopy.
